# The role of ginseng derivatives against chemotherapy-induced cardiotoxicity: A systematic review of non-clinical studies

**DOI:** 10.3389/fcvm.2023.1022360

**Published:** 2023-02-09

**Authors:** Arezoo Moini Jazani, AmirAhmad Arabzadeh, Hamed Haghi-Aminjan, Ramin Nasimi Doost Azgomi

**Affiliations:** ^1^Traditional Medicine and Hydrotherapy Research Center, Ardabil University of Medical Sciences, Ardabil, Iran; ^2^Department of Surgery, School of Medicine, Ardabil University of Medical Sciences, Ardabil, Iran; ^3^Pharmaceutical Sciences Research Center, Ardabil University of Medical Sciences, Ardabil, Iran

**Keywords:** cardiotoxicity, cisplatin, doxorubicin, ginseng, systematic review

## Abstract

**Aims:**

Although chemotherapy agents are used to treating cancers, they have serious side effects, like their harmful effects on the cardiovascular system, limiting the clinical use of these chemotherapy agents. This study aimed to systematically investigate the potential role of ginseng derivatives in the prevention of chemotherapy-induced cardiac toxicity.

**Methods:**

This systematic review was performed according to PRISMA guidelines strategy in databases till August 2022. First, identify studies related to using search terms in titles and abstracts. After studying and screening 209 articles, 16 articles were selected in this study according to our inclusion and exclusion criteria.

**Results:**

According to the findings of this study, ginseng derivatives showed significant changes in biochemical, histological, and heart weight loss, as well as a reduction in mortality, which occurred in the groups treated with chemotherapy agents compared to the control groups. Co-administration of ginseng derivatives with chemotherapy agents inhibited or reversed these changes to near-moderate levels. The protective effects of ginseng derivatives can be due to their anti-inflammatory, anti-oxidant, and anti-apoptotic action.

**Conclusion:**

This systematic review shows evidence that concomitant administration of ginseng derivatives improves chemotherapy-induced cardiac toxicity. However, for better conclusions about the practical mechanisms of ginseng derivatives in reducing the cardiac toxic effects of chemotherapy agents and evaluating the efficacy and safety of the compound simultaneously, it is necessary to design comprehensive studies.

## 1. Introduction

Cancer is caused by uncontrolled cell proliferation that has both benign and malignant types (1, 2). Numerous factors such as genetics, radioactivity, toxins, chemicals, and too much sunlight can cause cancer (3). Cancer is the second leading cause of death after cardiovascular disease and both are increasing (4). Cancer therapies include surgery, chemotherapy, radiotherapy, cryotherapy, targeted treatments, biological therapies, and immunotherapy (5). Although chemotherapy is a systemic therapy that is highly effective in treating and fighting cancer, it leads to changes in the body’s natural homeostasis and numerous side effects (6). Cases such as intolerance to chemotherapy agents, increasing resistance to chemotherapy agents, reduced therapeutic effects, and severe side effects lead to a reduction and limitation of chemotherapy in clinical use (7–9). Chemotherapy agents’ side effects include allergic reactions, nausea, and vomiting. Moreover, chemotherapy agents induced toxicity of various organs and tissues such as the heart, kidney, liver, gastrointestinal, nervous, etc. (10, 11). Complications of chemotherapy on the cardiovascular system include myocarditis, hypertension, acute or chronic heart failure, and dysrhythmia (7–9). Although cardiac oxidative stress has been suggested as an essential hypothetical mechanism in chemotherapy-induced myocardial infarction, its exact mechanism remains unclear (12, 13). On the other hand, various studies have suggested activating inflammation and apoptosis pathways by increasing reactive oxygen species (ROS) (14, 15). Due to chemotherapy-induced heart damage, using different methods to reduce heart damage is a significant challenge.

Ginseng is the root of the plant Panax species. This plant includes different types such as Panax ginseng Meyer (P. ginseng; Korean ginseng), Panax notoginseng (Chinese ginseng), Panax japonicum (Japanese ginseng), Panax quinquefolius (American ginseng), and Panax vietnamensis (Vietnamese ginseng). The clinical history of the use of ginseng worldwide for the treatment of various diseases such as improving physical function, immune function, exercise performance, reducing stress, and aging dates back more than 2,000 years ago (16).

Approximately 40 types of active ginseng active ingredients have been identified and isolated as ginsenoside (17). Ginsenosides, and triterpene saponins, are the most important components of ginseng’s active ingredients. Much of Panax ginseng literature focused on ginsenosides’ medicinal properties (18). Ginsengoids include a variety of such as protopanaxatriol type (ginsenoside Re, Rf, Rg1-2, Rh1), protopanaxadiol type (Ginsenoside Ra1-3, Rb1-2, Rc, Rd., Rg3, Rh2-3), ocotillo type (Makonoside-Rs), and oleanolic acid type (ginsenoside Ro) (19). Numerous studies on ginsenosides have shown that they have beneficial effects, such as anti-oxidant, anti-tumor, anti-diabetic, anti-aging, and organ-protective effects (20). Ginseng Panax and ginsenosides are usually well-tolerated and have low toxicity effects that are reversible. Various studies have been performed on different ginsenosides to investigate their effects and other mechanisms in chemotherapy-induced heart damage (21).

Various studies have shown, patients treated with chemotherapy have a lower capacity for anti-oxidant (22, 23). Therefore, it is assumed using some material such as ginseng and its derivatives, which have anti-oxidant properties can reduce the production of free radicals. Also, various studies have reported heart damage induced by chemotherapy agents (24–26). Considering, multiple studies have pointed to the protective effect of ginseng and its derivatives (27, 28). In the present systematic study, we investigate the role of ginseng and its derivatives in cardiac toxicity caused by chemotherapy. To investigate this, the present study was performed based on a comprehensive search of the role of ginseng and its derivatives in chemotherapy-induced cardiotoxicity. Attempts were also made to answer the following questions. Mechanisms that cause chemotherapy agents-induced cardiac toxicity? The role of ginseng or its derivatives during chemotherapy-induced heart toxicity? And the mechanisms of how ginseng or its derivatives play its role?

## 2. Methodology

Following the criteria outlined in the Preferred Reporting Items for Systematic Reviews and Meta-Analyses (PRISMA) standard, a comprehensive and systematic search was carried out (29). The review process was further organized using a PICO framework (29), which included participants (P): *in-vivo* or *in-vitro* studies with cardiac toxicity; intervention (I): *in-vivo* or *in-vitro* participants who receiving chemotherapy agents, or ginseng or its derivatives alone or in combination; Comparison (C): The participant that received chemotherapy compart group receives nothing and the participant in the group received chemotherapy agents and ginseng or its derivatives compared with the group receiving chemotherapy agents; outcomes (O): There were two critical outcomes: (1) Changes were brought about in the heart’s cells and tissue after chemotherapy treatment compared to control or untreated groups, and (2) changes were brought about in the heart’s cells and tissue after combination therapy of chemotherapy and ginseng or its derivatives compared to chemotherapy agents treatment alone.

### 2.1. Search strategy

We conducted a thorough and systematic search of the relevant published literature using various online databases, such as Scopus, PubMed, Web of Sciences, Embase, and Google Scholar up to August 2022. The search keywords were chosen based on the present study aims and presented in Supplementary file.

### 2.2. Inclusion and exclusion criteria

Two reviewers (HHA and RND) determined the eligibility criteria for each study that was included. All studies included in this systematic review met the following inclusion criteria: (1) Full-text papers published in English. (2) All observational studies were relevant to our objectives (*in-vivo* or *in-vitro*). From the included studies, the studies that meet the present exclusion criteria including (1) reviewed articles; (2) case reports; (3) posters; (4) book chapters; (5) letters to the editor; (6) oral communications; and (7) the articles which were not available, were excluded.

### 2.3. Study selection

The original articles according to present inclusion and exclusion criteria were chosen to be examined in this systematic review.

### 2.4. Data collection and quality assessment

Two authors (HHA and RND) extracted the following characteristics: the first author’s name, publication year, models, chemotherapy agents, protocol, and outcome, co-administration of ginsengs, treatment protocol, and outcome.

The Newcastle-Ottawa scale (NOS) was used to evaluate the quality of studies while taking into account three crucial factors: study group selection, adjustment for confounding variables, and outcomes evaluation (30). Results of the study quality assessment are shown in Table 1. The NOS scale in the included studies ranged from 7 to 8 stars.

**Table 1:** Quality assessment of case–control included in this systematic review.

Case–control studies	Is the case definition adequate?	Representativeness of the cases	Selection of controls	Definition of controls	Study controls for race/ethnicity, age, multivitamin supplementation, smoking	Study controls for any additional factor	Ascertainment of exposure	The same method of ascertainment for cases and controls	Non response rate	Total score
Jing Xing et al., 2019 (21)	*	*	*	*	*	ND	*	*	*	8
Zhang et al., 2017 (31)	*	*	*	*	ND	ND	*	*	*	7
FU et al., 2013 (32)	*	*	*	*	*	ND	*	*	*	8
Li et al., 2017 (33)	*	*	*	*	*	ND	*	*	*	8
Liu et al., 2008 (34)	*	*	*	*	*	ND	*	*	*	8
Jin Jang et al., 2019 (35)	*	*	*	*	*	ND	*	*	*	8
Wang et al., 2012 (36)	*	*	*	*	*	ND	*	*	*	8
Wang et al., 2015 (37)	*	*	*	*	*	ND	*	*	*	8
Meng Xu et al., 2018 (38)	*	*	*	*	*	ND	*	*	*	8
Sheng You et al., 2005 (39)	*	*	*	*	*	ND	*	*	*	8
Zhu et al., 2017 (25)	*	*	*	*	*	ND	*	*	*	8
Qiu et al., 2022 (40)	*	*	*	*	ND	ND	*	*	*	7
PI et al., 2021 (41)	*	*	*	*	ND	ND	*	*	*	7
Al-Kuraishy et al., 2022 (42)	*	*	*	*	*	ND	*	*	*	8
Akeel et al., 2022 (43)	*	*	*	*	*	ND	*	*	*	8
Hou et al., 2022 (44)	*	*	*	*	*	ND	*	*	*	8

According to the Newcastle-Ottawa Scale (NOS) criteria; ND, no description.

## 3. Results

### 3.1. Search results

**Figure 1:**
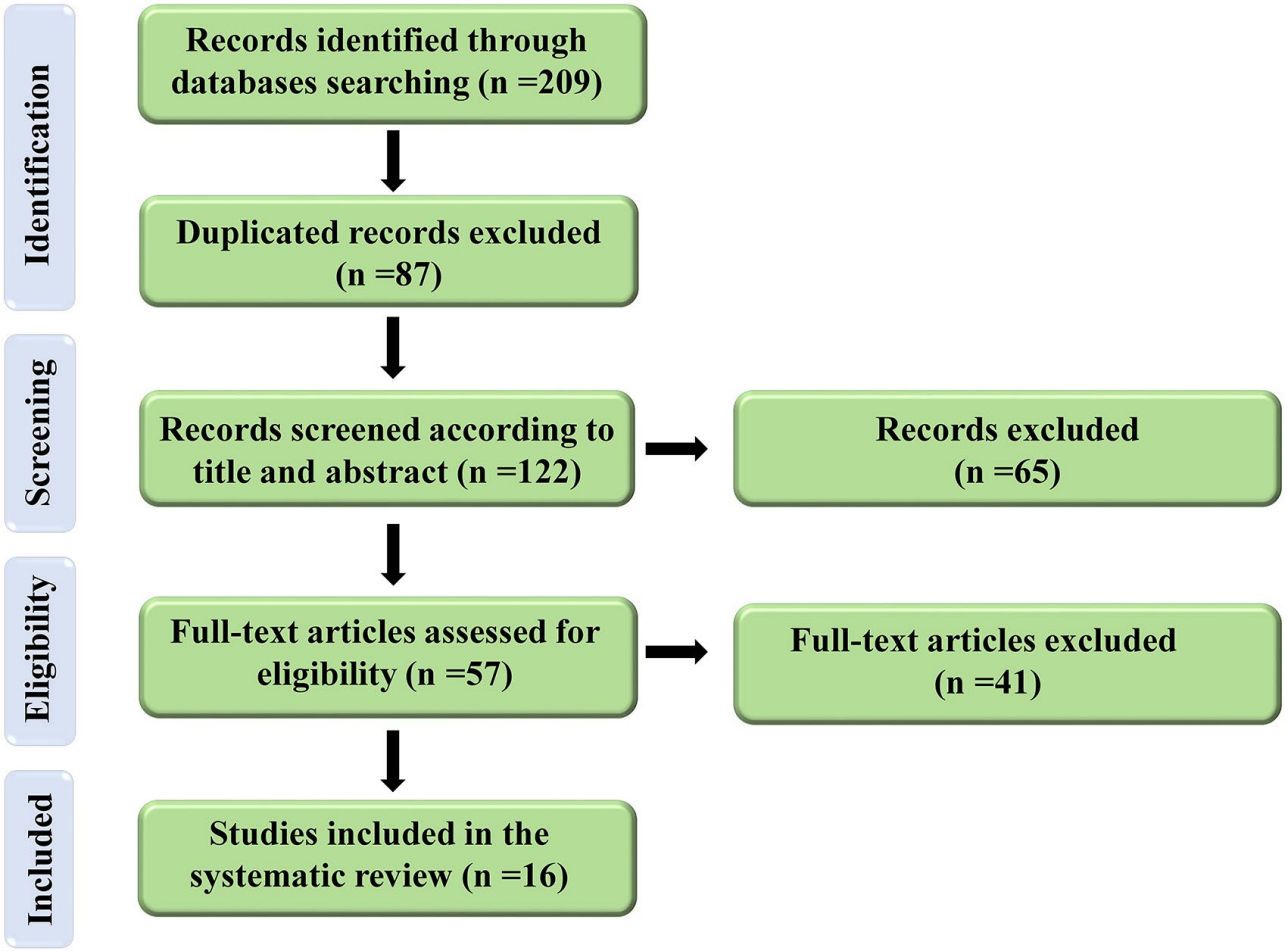
Flow diagram of the selection process for the present study.

The initial search on electronic databases yielded 209 which 87 duplicated articles were removed. Of those 122 articles screened in the title and abstract and 65 articles were excluded. Finally, 57 full-text of articles were screened and 16 articles were eligible to include in the present study. The flow diagram of the search strategy was illustrated in Figure 1.

### 3.2. Data extraction

Table 2 displayed more data from each article, these data were extracted by HHA and RND. Any discrepancies were discussed and agreed upon with the third author AMJ.

**Table 2:** The characteristics of included studies.

Authors name & year	Models	Chemotherapy drug (dosage) & route of administration	Chemotherapy outcome	Ginseng (derivatives) & dosage & route & duration of administration	Ginseng (derivatives) outcome
Jing Xing et al., 2019 (21)	*In-vivo*/Mice	Cisplatin (3 mg/kg) & *ip* on 7th, 9th, 11th, 13th day & 1 h after administration of PQS	↑ CK, ↑ CK-MB, ↑ cTnT, ↑ Degeneration in cardiac muscle fibers, ↓ GSH, ↓ SOD, ↑ MDA, ↑ TNF-α, ↑ IL-1β, ↑ LDH, ↑MPO, ↑ COX-2 level, ↑ iNOS level, ↑ ALT, ↑ p-NF-κB, ↑ p-IκBα, ↑ p-IKKα, ↑ p-IKKβ, ↑ Bax, ↑ Bad, ↑ Caspase-3, ↑ Caspase-8, ↑ Caspase-9, ↓Bcl-2 level, ↑ p-PI3K, ↓ p-Akt and ↑ GSK-3β	Panax quinquefolius & 125 mg /kg & orally & 15 days & from the start of experiment	↓ CK, ↓ CK-MB, ↓ cTnT, ↑ SOD, ↑ GSH, ↓ MDA, ↓ TNF-α, ↓ IL-1β, ↓ LDH, ↓ MPO, ↓COX-2 level, ↓ iNOS level, ↓ALT, ↓ p-NF-κB, ↓ p-IκBα, ↓ p-IKKα, ↓ p-IKKβ, ↓ Bax, ↓ Bad, ↓ Caspase-3, ↓Caspase-8, ↓Caspase-9, ↑ Bcl-2 level, ↓ p-PI3K, ↑ p-Akt and ↓ GSK-3β
				Panax quinquefolius & 250 mg /kg & orally & 15 days & from the start of experiment	↓ CK, ↓ CK-MB, ↓ cTnT, Normal myocardial morphology structure, ↑ SOD, ↑ GSH, ↓ MDA, ↓ TNF-α, ↓ IL-1β, ↓ LDH, ↓ MPO, ↓ COX-2, ↓ iNOS, ↓ ALT, ↓ p-NF-κB, ↓p-IκBα, ↓ p-IKKα, ↓ p-IKKβ, ↓ Bax, ↓ Bad, caspase-3, ↓ caspase-8, ↓ caspase-9, ↑Bcl-2 level, ↓ p-PI3K, ↑ p-Akt and ↓ GSK-3β
Zhang et al., 2017 (31)	*In-vitro*/H9C2 cells	Doxorubicin (1 μM/L–5 mM/L) & 24 h	DOX inhibited the growth of H9C2 cells, ↑ DNA fragmentation, ↑ changes in heterogeneous intensity, Chromatin condensation, & Fragmentation, ↑ Caspase-3/7 & 8 activity, ↑Cleaved Caspase-3, ↑Cleaved PARP protein, ↑ Bax expression, ↓Bcl-2 expression, ↑ Expression of CYP1A1, CYP1A2 & AhR genes, ↑ CYP1A1 luciferase reporter activity	Ginsenoside Rb1 & 50 μM & 6 h before doxorubicin administration	↑ Cell viability and ↓ DNA fragmentation, ↓ morphological changes
				Ginsenoside Rb1 & 100 μM & 6 h before doxorubicin administration	↑ Cell viability, ↓ DNA fragmentation, ↓ morphological changes, ↓ luciferase reporter activity
				Ginsenoside Rb1 & 200 μM & 6 h before doxorubicin administration	↑ Cell viability (highest effect), ↓ DNA fragmentation (highest effect), ↓ morphological changes, ↓ Caspase-3/7 and 8 activity, ↓ cleaved caspase-3, ↓cleaved PARP protein, not restored to Normal levels Cyt. C, Bax expression & Bcl-2 expression, ↓ induction of CYP1A1, CYP1A2, AhR mRNA and CYP1A1, CYP1A2 protein, ↓ luciferase reporter activity, inhibited the ability of Rb1 to decrease the induction of CYP1A and caspase-3 by transfection with AhR siRNA or AhR antagonist CH-223191
				Ginsenoside Rb1 & 400 μM & 6 h before doxorubicin administration	↑ Cell viability, ↓ DNA fragmentation, ↓ morphological changes and ↓ luciferase reporter activity
Fu et al., 2013 (32)	*In-vivo*/Mice	Doxorubicin (20 mg/kg) & *ip* & one dose & (acute)	↓ Survival time, All animals succumbed at the end of the experiment	Ocotillol (American Ginsengs) & 10 mg/kg/daily & 10 days & 24 h prior to doxorubicin injection	↑ Survival time, 2 of 10 animals remained alive at the end of the experiment
		Doxorubicin (3 mg/kg) & *ip* & six dose & (chronic)	↑ CK & CK-MB, ↓ GSH, ↑ MDA, Disorganization of myofibrillar arrays and cytoplasmic vacuolization, ↓ WBC	Ocotillol (American Ginsengs) & 10 mg/kg/daily & 8 days & 24 h prior to doxorubicin injection	↓ CK, ↓CK-MB, ↑ GSH, ↓ MDA, ↓ histopathological changes, ↑ WBC
				Ocotillol (American Ginsengs) & 10 mg/kg/daily & 8 days & 24 h prior to doxorubicin injection	↓ CK, ↓CK-MB, ↑ GSH, ↓ MDA, ↓ histopathological changes, ↑ WBC
Li et al., 2017 (33)	*In-vivo*/*Rat*	Doxorubicin (20 mg/kg) & *ip*	↑ LDH, ↑CK, ↑ CK-MB, ↑ Expressions of a-SKA, b-MHC genes, ↑ mRNA expression of Bax, ↑ Caspase 3, ↑ Caspase 9 protein expression, ↑ ROS, ↑ Ca^2+^ overload, ↓ATP production, ↓ MMR capacity, ↑ mtDNA, ↓ Caspase 3/7 activity, ↑ Mitochondrial membrane depolarization	Rg3 & 10 mg/kg & daily & orally & 14 days	↓ LDH, ↓CK, ↓ CK-MB, ↓ expressions of a-SKA, b-MHC genes, ↓ mRNA expression of Bax, ↓ Caspase 3, ↓ caspase 9 protein expression, ↓ ROS, ↓ Ca^2+^ overload, ↑ATP production, ↑ MMR capacity, ↓ mtDNA, ↑ Caspase 3/7 activity, ↓ mitochondrial membrane depolarization
	*In-vivo*/*Mice*	Doxorubicin (3 mg/kg) & *ip* & every 3 days & 4 times	↑Serum LDH, CK, CK-MB levels, ANP, & BNP, ↑Expressions of ANP, ANF, BNP, a-SKA, & b-MHC genes, ↓Heart volume, ↓Heart weight, ↓Ratio of heart weight to shank bone length, ↑Edema & cavitation, ↑Expression of Bax, ↓Expression of Bcl-2, ↑Caspase-3, & -9 protein expression, ↓Mitochondrial drill arrays, & swollen mitochondria, ↑ ROS level, ↑Ca^2+^ overload, ↓ATP content, ↓MMR capacity, ↓ OXPHOS complexes, ↓UCP3, ↓ATP5D, ↑mtDNA copy number, ↓Tumor weight	Rg3 & 10 mg/kg & daily & orally & 14 days	↑Serum LDH, CK, CK-MB levels, ANP, & BNP, ↓Expressions of ANP, ANF, BNP, a-SKA, & b-MHC genes, ↑Heart volume, ↑Heart weight, ↑Ratio of heart weight to shank bone length, ↓Edema & cavitation, ↓Expression of Bax, ↑Expression of Bcl-2, ↓ Caspase-3, & -9 protein expression, ↑Mitochondrial drill arrays, & swollen mitochondria, ↓ ROS level, ↓ Ca^2+^ overload, ↑ ATP content, ↑ MMR capacity, ↑ OXPHOS complexes, ↑ UCP3, ↑ ATP5D, ↓ mtDNA copy number, ↑ Tumor weight
	*In-vitro*/H9C2 & 4T1b & MDA-MB-231 cells	Doxorubicin (1–3 μM/L)	↓Cell viability ↓Caspase 3/7 activity, ↑cell hypertrophy, ↑ subcellular organelle damage, ↑ROS generation, ↑Ca^2+^ overload, ↑Mitochondrial membrane depolarization, ↓Basal respiration, ↓ATP production, ↑Non mithicondrial respiration, ↓Maximal respiration, ↓MMR capacity, ↓ Mitochondrial spare respiratory capacity, ↓OXPHOS complexes protein levels, ↓Cyc-C & UCP3 protein levels	P-Rg3 & 10 mg/kg & daily & orally & 14 days	↑ATP production, ↑MMR capacity, ↑OXPHOS complexes, ↑UCP3, ↑ ATP5D, ↓mtDNA, ↑Caspase 3/7 activity, ↓Cell hypertrophy, ↓Subcellular organelle damage, ↓ Mitochondrial ROS generation, ↓ Mitochondrial membrane depolarization, ↑ Caspase 3/7 activity in 4T1b cells & ↓Tumor weight
Liu et al., 2008 (34)	*In-vivo*/*Mice*	Doxorubicin (20 mg/kg) & *ip* & single dose	↑ LDH, ↑ CK & ↑ CK-MB, loss of myofibrils, myocardial rupture and vacuolization, ↓ SOD, CAT, & GPx activity	Panax notoginseng saponins (PNS) & 100 mg/kg & ig & daily & from the start of experiment	↓ LDH, ↓ CK, ↓ CK-MB, ↓ morphological changes & ↑ CAT
				Panax notoginseng saponins (PNS) & 100 mg/kg & ig & daily & 5 days before doxorubicin administration	
Jin Jang et al., 2019 (35)	*In-vivo*/*Rat*	Doxorubicin (20 mg/ kg) & subcutaneously & on 8th, 9th, day	↓Activity of SOD, CAT, & GPx, ↑ MDA level, ↑cTnI, & MPO activity, ↑ Interstitial edema, ↑ Hemorrhage, ↑Loss of myofibrils & ↑ Fiber disorganization	Korean Red Ginseng (KRG) & 250 mg/kg & ig & 10 days+ doxorubicin on 8th, 9th, day	↑SOD, ↑CAT, ↑GPx, ↓MDA, ↓cTnI, ↓MPO, ↓Loss of myofibrils, ↓Fiber disorganization and better general architecture of cardiac tissue
				Korean Red Ginseng (KRG) & 500 mg/kg & ig & 10 days+ doxorubicin on 8th, 9th, day	
Wang et al., 2012 (36)	*In-vivo*/*Mice*	Doxorubicin (3 mg/kg) & *ip* & 6 times	↑Serum CK, ↑ LDH, ↑ AST, ↓SOD, ↓GSH, ↓CAT in heart tissue, ↑MDA, ↑Histopathological changes of myocardial cells	20(S)-ginsenoside Rh2 (Rh2) & 5, 10, 20 mg/kg & ig daily & 8 doses & at 24 h before doxorubicin injection	↓Serum CK, ↓ LDH at the dose of 10 and 20 mg/kg, ↓AST with different dose of Rh2, ↑SOD, ↑CAT, ↑GSH, ↓MDA, ↓ Histopathological changes of myocardial cells
	*In-vitro*/*H9C2* cells	Doxorubicin (1–2 μM)	↓Growth of H9C2 cells	20(S)-ginsenoside Rh2 (Rh2) & 5, 10, 20 μM & 2 h before doxorubicin	↑Cell viability in 5, 10, 20 μM concentration	
	*In-vitro*/*A549* cells	Doxorubicin (1–2 μM)	Antitumor activity	20(S)-ginsenoside Rh2 (Rh2) & 5, 10, 20 μM & 2 h before doxorubicin	Synergistically increases antitumor activity
Wang et al., 2015 (37)	*In-vivo*/*Rat*	Doxorubicin (15 mg/kg) & *ip* & single dose	↑ Endothelium dysfunction	Ginsenoside Rg3 (Rg3) & 10, 20, 40 mg/kg & ip & at 1 h after doxorubicin injection & 14 days	↓ Endothelium dysfunction
	*In-vitro*/*CMEC* cells	Doxorubicin (1 μM/l)	↓ Cell viability, ↑ LDH, ↓ eNOS, ↑ ROS, ↑MDA, ↓ SOD, ↓ SOD-1/GPx-1 mRNA expression, ↓ SOD-2/GPx-1 mRNA expression, ↑ Fas m RNA expression, ↑ Bax/ Bcl-2, ↑Annexin v binding to CMEC, ↑Ca^2+^, ↓ Nrf2, ↓ HO-1, ↑ Keap1, ↓AKT T308, ↑ ICAM-1, ↑VEGF, ↑ TIMP-1, ↑ TGFβ	Ginsenoside Rg3 (Rg3) & 10^−6^, 10^−5^, 10^−4^ M & 24 h	↑ Cell viability in 10^−5^, 10^−4^ M, ↓ LDH from 10^−6^ to 10^−4^ M, ↑ eNOS, ↓ ROS in 10^−5^, 10^−4^ M, ↓MDA, ↑ SOD, ↑SOD-1/GPx-1 mRNA expression in 10^−5^, 10^−4^ M, ↑SOD-2/GPx-1 mRNA expression in 10^−4^ M, ↑ Fas m RNA expression, ↑ Bax/ Bcl-2 in10^−5^, 10^−4^ M, ↓ Annexin v binding to CMEC, ↓ Ca^2+^, ↑ Nrf2 and ↑ HO-1 in 10^−5^, 10^−4^ M, ↓ Keap1, ↑ AKT T308 in 10^−5^, 10^−4^ M, ↓ ICAM-1, ↓ VEGF and ↓ TIMP- in 10^−5^, 10^−4^ M 1, ↓ TGFβ
Meng Xu et al., 2018 (38)	*In-vivo*/*Mice*	Doxorubicin (6 mg/kg) & *ip* & single dose & every 3 days & 4 times	↑ Myofibrillar degeneration & disruption, ↑ Cardiac fibrosis, ↑Conversion of LC3A to LC3B, ↑ Expressions of ATG5 & sequestosome 1 (P62), ↑ ER dilation, ↑ cleaved ATF6 & IRE1 by protein expression, ↓ Expressions of XBP1s, ↓ GFAT1, ↑ TIF1, ↑ mRNA translation & ↓ the expression of GRP78 (ER chaperone), ↑ Expression of phosphorylated ribosomal protein S6 kinase beta-1 (p-P70S6K)	Ginsenoside Rg1 & 50 mg/kg & i.g & 7 days before doxorubicin injection	↓ Myofibrillar degeneration and disruption, ↓ Cardiac fibrosis, Suppress of conversion of LC3A to LC3B, ↓ Expressions of ATG5 & sequestosome 1 (P62), ↓ ER dilation, ↓ Cleaved ATF6 & IRE1 by protein expression, ↑ Expressions of spliced X-box binding protein 1 (XBP1s), ↑ Glutamine fructose-6-phosphate amidotransferase (GFAT1), ↓ TIF1, ↓ mRNA translation, ↑ The expression of GRP78 (ER chaperone), ↓ Expression of phosphorylated ribosomal protein S6 kinase beta-1 (p-P70S6K)
Sheng You et al., 2005 (39)	*In-vivo*/*Rat*	Doxorubicin (2.5 mg/kg) & *ip* & 6 times	↓ Heart weight, ↓ heart weight/body weight, ↓ Systolic and diastolic arterial pressure, ↓ Synthesis rates of DNA, RNA and protein, ↓ GPx, ↓ SOD, ↑ MDA	PG (Panax ginseng treated) & 5 g/kg & orally & daily & 30 days & alternating with adriamycin injections.	↓ Peritoneal fluid, ↓ Mortality rate, ↑ Heart weight, NS heart weight/body weight, NS systolic & diastolic arterial pressure, ↑ Synthesis rates of DNA, RNA & protein, ↑ GPx, ↑ SOD, ↓ MDA
Zhu et al., 2017 (25)	*In-vivo*/*Mice*	Doxorubicin (15 mg/kg) & *ip* & single dose	↑ LDH, ↑ CK-MB, ↑ Infiltration of inflammation, ↑ Fibrosis of the heart, ↑ Cyt. C, ↑ Cleaved caspase-3, ↓ Phosphorylation of Akt & Erk, ↓ Bcl-2 & Bax ratio	Ginsenoside Rg & 180 mg/kg & day & orally & 1 week before doxorubicin injection	↓ LDH, ↓ CK-MB, ↓ Infiltration of inflammation, ↓ Fibrosis of the heart, ↓ Cyt. C, ↓ Cleaved caspase-3, ↑ Phosphorylation of Akt and Erk, ↑ Bcl-2 & Bax ratio
Qiu et al., 2022 (40)	*In-vitro*/*H9C2 cells*	Doxorubicin (2.5, 5, 10, 15, and 20 μM) & 24 h	↓ Cell viability, ↑ ROS, no significantly different Akt, ↑ p53 and p-p53 expression	Ginsenoside Rg2 & 100, 200, 250, 300, 350, and 400 μM & 24 h prior to doxorubicin 24 h	↑ Cell viability, ↓ Apoptotic rate in 200 μM and 250 μM, ↓ ROS, no significantly different Akt, ↑ p-Akt/Akt, ↓ p53 expression, not significantly inhibit p-p53, upregulates Akt phosphorylation
PI et al., 2021 (41)	*In-vitro*/*H9C2 cells*	Adriamycin (2.67 μmol/L) & 45 h	↑ Injured H9C2 cells, ↑ Inflammatory cytokines, ↑ Apoptosis rate, ↓ Expression of miR-130b, ↑PTEN	Ginsenoside Rb1 & 0, 25, 50, 100, and 200 μM & 6 h	Ameliorate the proliferation of injured, ↓Inflammatory cytokines, ↓IL-1β, ↓IL-6, ↓TNF-α, ↓f P53, ↓Bax, ↓ cleaved-caspase3, ↑Bcl-2, ↑ Ki67, ↑PCNA, ↓PTEN, ↑p-PI3K, ↑p-AKT, ↑ Expression of miR-130b
Al-Kuraishy et al., 2022 (42)	*In-vivo*/*Rats*	Doxorubicin (15 mg/kg) & ip & single dose & in the day eight	↓GP serum level, ↑ LPO, ↑ MDA levels, ↑ cTnI, ↑BNP, Caspase-3, ↑TNF-α levels, ↑Congested vessels, Extravasation of red blood cells, Cytoplasmic vacuolations, Edema, Decreased nuclei, Fragmentation with necrosis, Loss of muscle fiber striation	Panax ginseng (100 mg/kg) & daily & orally & 10 days & before doxorubicin injection	↑ GP, ↓ MDA levels, ↓ n cTnI, ↓ BNP, ↓ Caspase-3, ↓ TNF-α, Ameliorate myocardial damage
Akeel et al., 2022 (43)	*In-vivo*/*Rats*	Doxorubicin (20 mg) & ip & single dose & in the day eight	↑ BNP, ↑MDA, ↑ LPO, ↑TNF-α, ↑ Caspase-3, ↓GSH, ↑ Dilatation of coronary arteries & congestion with RBC, ↓Number of nuclei of myofibrils	Panax ginseng (100 mg/kg) & daily & orally & 10 days	↓ cTnI, ↓ LPO, ↓ MDA, ↓ DIC ↑ GSH, ↓TNF-α, ↑ Caspase level, ↓ edema, ↓ Artery irregularity, ↓Heart fibers confusion
Hou et al., 2022 (44)	*In-vivo*/*Mice*	Doxorubicin (2 mg/kg) & *ip* & every other day	↓Tumour weight, ↑ Histological damage with congestion of heart tissue, ↑Cleaved caspase 3, ↑P53, ↑SMAC, ↑TRAIL R2, ↓Bcl-2, ↓Bcl-x, ↓ Catalase, ↓ HO-2, ↓ HSP27, HSP60, ↓ XIAP, ↑ TLR2, ↑ TLR6, ↑TLR7, ↑ TLR8, ↑TLR11, ↑TLR 13,	Ginsenoside Rh2 & 20, 30 mg/kg & injected every other day & 3 weeks	↓ Cardiac histopathological Changes, ↓Apoptosis & necrosis, ↓Fibroblast to myofibroblast transition, ↓ Endothelial-mesenchymal transition, ↓Cleaved caspase 3 ↓Expression of IL-1β, ↓TNF-α, ↓IL-6, ↓TLR2, ↓ TLR6, ↓TLR7, ↓TLR8, ↓TLR11, ↓TLR 13, ↓proteins smad2, ↓ smad3
	*In-vitro*/*H9C2 cells*	Doxorubicin (100 nM) & 7 days		Ginsenoside Rh2 & 2.5, 5, and 10 μg/ml after doxorubicin	

↑, Increase; ↓, Decease; &, and; ip, Intraperitoneal; po, Per os; sc, subcutaneously; CK-MB, Creatine kinase-myocardial bound; CK, Creatine kinase; LDH, Lactate dehydrogenase; MDA, Malondialdehyde; SOD, Superoxide dismutase; ROS, Reactive oxygen species; GSH, Glutathione; Ca^2+^, Calcium ion; GPx, Glutathione peroxidase; cTnT, Cardiac troponin T; Bcl-2, B-cell lymphoma 2; Bax, BCL2-associated X protein; MPO, Myeloperoxidase; TNF-α, Tumor necrosis factor alpha; IL-1β, Interleukin 1 beta; Erk, Extracellular signal-regulated kinase; ATP, Adenosine triphosphate; ATG5, Autophagy related 5; P62, Sequestosome 1; ATF6, Activating transcription factor 6; XBP1s, X-box binding protein 1; GFAT1, Glutamine fructose-6-phosphate amidotransferase; TIF1, Transcriptional intermediary factor 1; GRP78, Glucose regulated protein-78; p-P70S6K, phosphorylated ribosomal protein S6 kinase beta-1; eNOS, Endothelial nitric oxide synthase; Nrf2, nuclear factor erythroid 2–related factor 2; HO-1, Heme oxygenase*-*1; Keap1, Kelch-like ECH-associated protein 1; ICAM-1, Intercellular Adhesion Molecule 1; VEGF, Vascular endothelial growth factor; Tissue inhibitor matrix metalloproteinase 1, TIMP-1, Tissue inhibitor matrix metalloproteinase 1; TGFβ, Transforming growth factor beta; AST, Aspartate aminotransferase; OXPHOS, Oxidative phosphorylation; MMR, mitochondrial maximal respiration; Cyt. C, Cytochrome c. Cyc-C, cytochrome complex; UCP3, Uncoupling Protein 3; MMR, mitochondrial maximal respiration; WBC, white blood count; COX-2, cyclooxygenase*-*2; iNOS, Inducible nitric oxide synthase; ALT, Alanine aminotransferase, p-NF-κB, Nuclear factor kappa-light-chain-enhancer of activated B cells; p-IκBα, nuclear factor of kappa light polypeptide gene enhancer in B-cells inhibitor, alpha; p-IKKα, Inhibitor of kappaB kinase alpha; p-PI3K, Phosphoinositide 3-kinase; GSK-3β, Glycogen synthase kinase-3 beta.

### 3.3. The role of ginseng derivatives against cardiotoxicity induced by doxorubicin, and cisplatin

#### 3.3.1. Doxorubicin

Doxorubicin, under the brand name Adriamycin, is a well-known anti-neoplastic anthracycline drug that is very effective in treating various cancers. These cancers include bladder cancer, lymphoma, breast cancer, Kaposi’s sarcoma, and acute lymphocytic leukemia (1, 45). Dose-dependent cardiotoxicity of this drug limits its clinical use of this drug (31). One of this drug’s most dangerous side effects is dose-dependent dilated cardiomyopathy, which leads to congestive heart failure (45).

Studies have shown that doxorubicin can cause some biochemical changes compared to controls. This drug can increase the level of ROS, catalase (CAT), malondialdehyde (MDA), tumor necrosis factor-alpha (TNF-α), plasma nitric oxide (NO), creatine kinase-MB (CK-MB), creatine kinase (CK), LV tissue O_2_, calcium (Ca^2+^), and adenosine triphosphate (ATP) and decrease anti-oxidant enzyme activity (CAT, SOD (Superoxide dismutase), GPx (glutathione peroxidase)), glutathione/glutathione-disulfide ratio (GSH/GSSG), high-density lipoprotein (HDL), total sulfhydryl groups (total-SH) level, and nonprotein sulfhydryl (NP-SH) groups level. The results of studies show that ginseng derivatives can reverse these biochemical changes caused by doxorubicin (21, 31–37, 40, 42, 43, 46). Previous studies demonstrated ginseng derivatives through several mechanisms can reduce cardiotoxicity including regulating intracellular ion channels (intracellular calcium homeostasis) (47), suppressing apoptosis by regulation of B-cell lymphoma 2 (Bcl-2) and caspase-3 (47), anti-oxidant actions *via* nuclear factor erythroid 2–related factor 2 (Nrf2) (48), also by increasing internal anti-oxidant enzymes and acting as a free radical scavenger regulating Akt/phosphoinositol-3-kinase (PI3K) pathway (49), and endothelial nitric oxide synthase (eNOS) (50). The current research demonstrates the effect of doxorubicin on the histological changes in various areas of the animal heart. Doxorubicin treatment causes disorganization of myofibrillar arrays and cytoplasmic vacuolization (32), loss of myofibrils, myocardial rupture, and vacuolization (34), histopathological changes of myocardial cells (36), endothelium dysfunction (37, 42–44). Moreover, it indicates the elevation of myofibrillar degeneration and disruption, cardiac fibrosis (38), inflammation infiltration, and the elevation of fibrosis of the heart (25). Mice treated with doxorubicin and ginseng derivatives together had less tissue damage or inhibited or suppressed the degenerative changes caused by doxorubicin (25, 33–39, 41–44). In addition, the results of this study showed that doxorubicin treatment increased mortality compared with the control group in the animals studied. Concomitant use of ginseng with doxorubicin significantly reduces mortality (32, 39). Furthermore, a review of these studies showed that doxorubicin reduced heart weight compared to controls. However, when co-administered with doxorubicin, ginseng derivatives significantly reduced heart weight than animals receiving doxorubicin (39). Cell studies suggest that ginseng derivatives *via* the aryl hydrocarbon receptor (AhR) could inhibit apoptosis (31, 37) and induce a reduction in cardiomyocyte damage and doxorubicin-induced apoptosis by decreasing caspases 3 and 8 activity. Ginsenoside Rb1 decreased the doxorubicin-induced expression of CYP1A1 and CYP1A2 (31). The cell study also showed that Ginsenoside Rg3 and (S)-GinsenosideRh220 could be identified as protective agents against doxorubicin-induced cardiotoxicity (increased free radical production) (33, 36). Ginseng improves management by improving mitochondrial function and metabolic activity, regulation of Ca^2+^ level (33), and decreased ROS production (anti-oxidant properties) can reduce cardiac toxicity and increase antitumor properties by doxorubicin (35–37, 40).

#### 3.3.2. Cisplatin

Cisplatin is a type of chemotherapy that is used to treat patients who have solid tumors. The significant adverse effect of cisplatin known as cardiotoxicity severely restricts its applications (51). Cardiotoxicity induced by cisplatin causes biochemical changes, including elevation of serum CK level increases, LDH activity, and heart troponin elevation (21). Moreover, cisplatin induces the elevation of superoxide radicals and malondialdehyde (MDA) (21). These biochemical changes were returned to normal levels when cisplatin and ginseng derivatives were used concomitantly. Cisplatin reduces the activity of SOD and the level of GSH, which is a concomitant treatment with ginseng derivatives, and returns the levels of these enzymes to normal. Degeneration changes in cardiac muscle fibers are seen in cisplatin consumption, which returns to normal with concomitant use with ginseng derivatives (21).

## 4. Discussion

**Figure 2:**
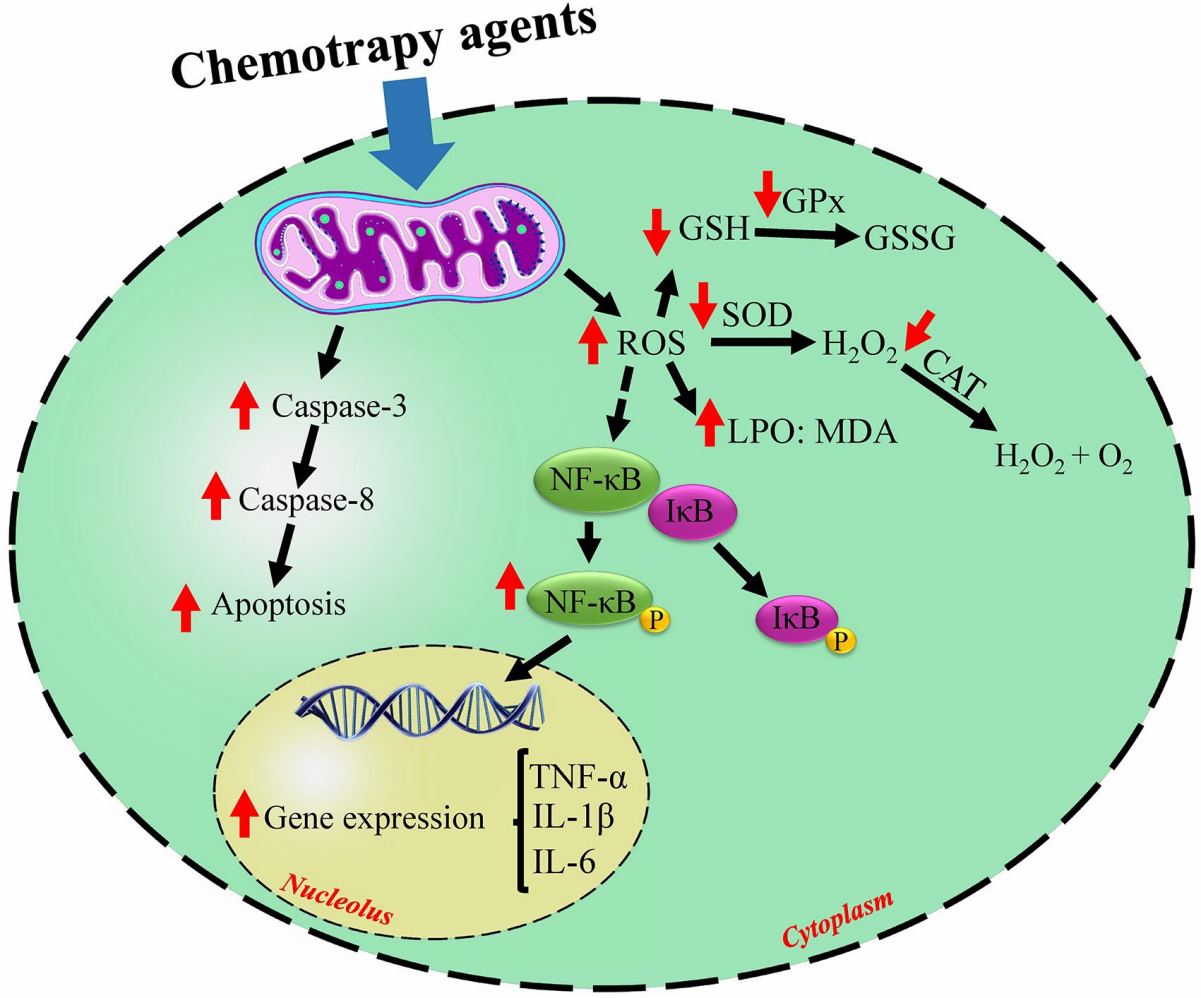
The general mechanisms of chemotherapy-induced cardiotoxicity. Chemotherapy agents induce free radicals which induce oxidative stress, trigger apoptosis, and inflammation. ↑ increased by chemotherapy; ↓ decreased by chemotherapy; ROS, reactive oxygen species; SOD, superoxide dismutase; CAT, catalase; GPx, glutathione peroxidase; MDA, malondialdehyde; LPO, lipid peroxidation; GSH, glutathione; GSSG, glutathione disulfide; NF-κB, nuclear factor kappa-β; TNF-α, tumor necrosis factor alpha; IL-6, interleukin 6; IL-1β, interleukin 1 beta.

Clinical use of doxorubicin and cisplatin as effective chemotherapy drugs is associated with various nonspecific adverse events such as cardiotoxicity, gastrointestinal problems, etc. (52). The main and exact cardiotoxic mechanisms of cisplatin and doxorubicin are not fully understood. Still, in, several studies, oxidative stress, inflammation, and apoptosis have been identified as the main mechanisms of the cardiotoxicity of these agents. Figure 2 illustrates the primary mechanisms that chemotherapy agents in this study employ.

The *Streptomyces peucetius* var. *caesius* microorganism produces doxorubicin as a secondary metabolite (53). Doxorubicin belongs to the family of anthracyclines (54). Doxorubicin is an effective agent in treating various cancers, especially pediatric, leukemia, and breast cancer (55, 56). This agent damages cell DNA by inhibiting the enzyme topoisomerase II (57).

Administration of anthracyclines above a dose of 400–700 mg/m^2^ leads to cardiac complications. 9% of people who receive chemotherapy with anthracyclines develop a decrease in left ventricular ejection fraction (EF) (58, 59).

Several mechanisms have been suggested for the cardiotoxicity of doxorubicin, including increased ROS production and decreased levels of anti-oxidants, as well as impaired intracellular Ca^2+^ regulation and apoptosis. As mentioned, one of the most sensitive systems in the body to chemotherapy agents is the cardiovascular system. Cardiac toxicity by chemotherapy agents can reduce the quality of life, impose high costs on the patient, and limit the clinical use of doxorubicin and cisplatin (40, 60). Ginsenoside Rg3 and Ginsenoside Rb1 are all examples of saponins that are derived from ginseng and have a variety of pharmacological activities. Some of these activities include the enhancement of detoxification and immunity, protection of the cardiovascular system, and the inhibition of the invasion, proliferation, and metastasis of cancer cells (61, 62).

The current study aimed to investigate the effects of cardiotoxicity complications caused by doxorubicin and cisplatin as well as the effects of concomitant administration of ginseng during chemotherapy. The results of the current systematic study showed that concomitant use of ginseng could reduce chemotherapy-induced cardiotoxicity. Findings from animal and cellular studies demonstrated ginseng might play a potential role in protecting the heart against the side effects of chemotherapy agents through various mechanisms, including reducing oxidative stress, inflammation, and apoptosis. Ginseng has also been shown to elevate the activity of anti-oxidant enzymes including SOD, CAT, and GPx. It also has anti-apoptotic, anti-inflammatory, and membrane-stabilizing properties (21, 35, 37, 39, 40, 43).

The following discussion investigates the properties of chemotherapy agents on heart cells and ginseng’s protective role.

### 4.1. Oxidative stress effects

When oxygen leads to the production of free radicals can have toxic effects. Under physiological conditions, the enzymatic and non-enzymatic anti-oxidant defense systems can strike a balance (63, 64). On the other hand, in pathological conditions such as toxicity, metabolic diseases, inflammation, and cancer, the formation of free radicals over the anti-oxidant system’s capacity can affect all organs in the body (65–69). Oxidative stress is induced through the elevation in free radicals such as hydroxyl (OH^−^), superoxide (O^2−^), singlet oxygen (^1^O_2_), and other secondary ROS as well as a decrease in anti-oxidant defense. Increasing the amount of oxygen free radicals disrupt the function of cellular carbohydrates, proteins, nucleic acids, and lipids, leading to disease (70). Chemotherapy-induced oxidative stress has been shown to have a mechanical role in myocardial dysfunction (71). Some ROS react strongly together with short half-lives and produce more potent, more stable, and more toxic free radicals (H_2_O_2_, HOCl, OCl^−^) (72). It can produce OH^−^ which is highly reactive and highly toxic. During times of oxidative stress, anti-oxidant enzymes like CAT, SOD, and GPx neutralize the free radicals that may threaten the tissues and cells in the body (73). When there is a decrease in the amount of anti-oxidant enzymes, this leads to an increase in the production of ROS as well as induced oxidative stress (74). These reactions persist on an intermittent basis and result in the production of more toxic species such as hydroxyl radicals (75). GSH is an extremely important tripeptide to shield cells from damage caused by free radicals. GSH can react with ROS, lowering the ROS concentration (76). Due to the fact that it is converted to GSH disulfide when subjected to oxidative stress, the level of GSH in the heart is reduced when these conditions are present. It is demonstrated that dysfunction of the cell can be attributed to the reaction of ROS with various components of the cell. By removing the ROS produced, anti-oxidants can have protective effects against doxorubicin-induced cardiomyopathy (54).

One possible hypothesis for chemotherapy agents induced cardiotoxicity is through elevation of the generation of free radicals (71). It has been observed that doxorubicin can transform into a semi-quinone radical *via* NADH dehydrogenase (complex I) of the mitochondrial electron transport chain (ETC) (77), NADPH-dependent cytochrome P450 reductases of the endoplasmic reticulum and nuclear envelope (78), and cytosolic xanthine oxidase (79). This semiquinone radical can auto-oxidize rapidly consuming molecular oxygen and lowering oxygen generation while enhancing superoxidase and creating lipid hydroperoxide after reacting with unsaturated fatty acids (80). The main mechanism of cisplatin-induced cardiac toxicity is oxidative stress. Cardiac dysfunction induced by cisplatin is related to mitochondrial membrane depolarization and ultrastructural abnormalities (81). O^2−^ is one of the ROS molecules that the SOD enzyme transforms into H_2_O_2_ (82, 83). Additionally, during the dysfunction of mitochondrial NADPH oxidases, non-radical ROS, such as hydrogen peroxide (H_2_O_2_) produced and transported to the cytoplasm by aquaporin. H_2_O_2_ has several purposes: (1) It produces the H_2_O and O_2_ from H_2_O_2_ by the enzyme CAT (84). (2) H_2_O_2_ produces OH through Fenton reactions and the Haber-Weiss network (85). (3) H_2_O_2_ produces 2H_2_O through GPx activity and GSH consumption (86).

Doxorubicin has the potential to raise the level of NO, which is normally present in only trace amounts in the cells of the heart. Within this framework, NO plays an important part in the cellular signaling processes that occur during pathological processes (87, 88). When NO combines with O^2−^, the result is a compound known as ONOO^−^, which is a potential free radical. ONOO^−^ has the potential to transform into NO^2−^, NO^3−^, and OH^−^. As a result, oxygen radicals can stimulate the generation of active nitrogen species (RNS). It has been revealed that doxorubicin causes DNA damage by elevating oxidative stress and lowering ADP-ribose polymerase (PARP) enzyme activity (89, 90). Doxorubicin increases LPO markers, including 4-HDA, TBARs, and MDA, resulting in damage and dysfunction of the cell membrane. Under these circumstances, extracellular ions, particularly Ca^2+^, rapidly enter the cells, leading to cell dysfunction and ultimately apoptosis. In addition, oxidative stress is made worse by the peroxyl radical, which is generated by LPO (70, 90). Ginseng is a powerful anti-oxidant with heart-protecting effects. Ginseng, directly and indirectly, reduces oxidative stress. As a direct anti-oxidant, Ginseng has been shown to scavenge free radicals. Consequently, it can reduce the LPO. Ginseng’s ability to regulate anti-oxidant defense also allows it to boost the efficiency of anti-oxidant enzymes like GSH, CAT, and SOD. Ginseng is a root that grows in Asia (91).

### 4.2. Inflammatory effects of cisplatin and doxorubicin, cardioprotective effects of ginseng

Inflammation is a protective reaction involving blood vessels, molecular mediators, and immune cells that is a component of the intricate biological response of body tissues to harmful stimuli, such as pathogens, irritants, or damaged cells (92, 93). The results of several studies indicate that heart damage caused by cisplatin and doxorubicin causes an imbalance in pro-inflammatory and anti-inflammatory cytokines (45, 94). Activating NF-κB is critical in the body’s reaction to inflammatory stimuli. The cytotoxic effects of chemotherapy agents are ultimately increased when the NF-κB pathway is stimulated, followed by an increase in pro-inflammatory cytokines. Additionally, they raise the expression levels of chemokines and other pro-inflammatory cytokines including IL-6, IL-1β, COX-2, and iNOS. These pro-inflammatory cytokines control the rate at which neutrophils penetrate the damaged site in the heart (92, 95, 96).

For many years ginseng used as a remedy for various ailments such as immune diseases, liver disease, and cancer, ginseng has been used for thousands of years in Asian societies, including China, Korea, and Japan (32). Ginsenosides are the active ingredients in ginseng and are responsible for most of its medicinal benefits. Ginsenosides engage in various activities, including neuroprotective, cardiac protective, and anti-cancer effects. There have been approximately 200 ginsenosides discovered up until this point. These have included major ginsenosides (such as Rc, Rd., Re, Rb1, Rb2, and Rg1) and minor ginsenosides (Rh1, Rh2, and Rg3). These ginsenosides are categorized into two important groups, such as protopanaxatriol (PPT) and protopanaxadiol (PPD), both of which have a major hydrophobic column of a four-ring steroid with sugar but differ in carbohydrates at positions C3, C6, and C20 (97). The result of the present study demonstrated cisplatin and doxorubicin can elevate inflammation in heart tissue.

Various studies show that ginseng strongly inhibits inflammation through the down-regulation of IL-6, IL-1β, and TNF-α (21). The results of several studies indicate that ginseng can down-regulated iNOS and COX-2, gene expression, inhibit IKKβ phosphorylation and NF-κB phosphorylation, and mitigate NF-κB DNA binding activity. Inhibition of IKKβ phosphorylation and increased IκBα activity suppress NF-κB pathway expression and ultimately reduce the production of inflammatory cytokines (21). Intercellular adhesion molecule (ICAM) is a surface protein that plays a significant role in the infiltration of leukocytes into injured areas of heart tissue. In 2015, Wang et al. (37) showed that ginseng could reduce ICAM marker which is enhanced by doxorubicin. On the other hand, the results of several histological studies showed a significant decrease in the infiltration of inflammatory cells into the site of damaged heart cells by doxorubicin following the use of ginseng (21, 31–33, 35, 36). In addition, it is reported that IL-6 stimulates the phosphorylation of JAK2 and STAT3 through the stimulation of TIMP-1 promotion by M1 macrophages (98). Gingsing through modulation of IL-6 and TIMP-1 modulates inflammation through the JAK2/STAT3/NF-κB pathway (99).

### 4.3. Apoptotic effects of cisplatin and doxorubicin, cardioprotective effects of ginseng

Apoptosis is a crucial regulatory system for cell death that is crucial for both cell death and the homeostasis of multicellular organisms (100, 101). When this cellular pathway is impaired, tissue diseases and malignancies can result because it is necessary for sculpting tissue, regulating cell populations, and killing damaged or altered cells (102, 103). Although the mechanisms of doxorubicin-induced cardiac toxicity have not been fully elucidated, the induction of cardiac apoptosis is one of the primary features of doxorubicin-induced cell damage (45, 94). Doxorubicin tends to build up not only in the nucleus but also in the mitochondria of affected cells. By releasing cytochrome c (Cyt. C) into the cytoplasm and thereby activating caspases, mitochondria are an essential component in the process of apoptosis, which occurs when cells die. Oxidative stress brought on by doxorubicin and abnormally high levels of calcium in the cell work together to trigger the release of Cyt. C and the beginning of the apoptotic pathway through the activation of caspase (25). A further mechanism by which doxorubicin may promote apoptosis is through its influence on mitochondrial topoisomerase II. In the presence of topoisomerase II, the drug doxorubicin will activate DNA response genes, and this will lead to the activation of apoptotic pathways. These properties cause significant alterations in DNA transcription, which, in turn, selectively affect mitochondrial biogenesis and oxidative phosphorylation in cardiomyocytes, which ultimately results in mitochondrial metabolic failure and oxidative stress. In addition, doxorubicin derivatives cause an increase in the release of cytokines because they accumulate in the inner membrane of mitochondria and disrupt the electron transport chain. In the current study, we discovered that doxorubicin meaningfully boosted the amount of Cyt. C that was released from mitochondria in the heart (25). Because of this, it seems reasonable to employ a strategy that targets the mitochondrial apoptotic pathway to prevent doxorubicin’s induction of cardiac toxicity (25). There have been 15 different agents, including anti-oxidants, angiotensin-converting enzymes, metal chelators, and beta-blockers, that have been used to inhibit doxorubicin-induced cardiac toxicity, and all of them have had some level of success. Herbal remedies are effective in avoiding the cardiac toxicity initiated by doxorubicin, according to recent research (25).

It has been demonstrated in some studies that the anti-apoptotic effect of Rg1 protects the heart from ischemic reperfusion injury as well as myocardial infarction (25).

The phosphorylation of Erk and Akt was increased as a result of Rg1 activity. Through Akt phosphorylation in endothelial cells, Rg1 stimulates the growth of new blood vessels. In response, Erk prevents apoptosis in endothelial cells by elevating the level of phosphorylation of Erk (25).

Both the mitogen-activated protein kinase (MAPK) pathway and the PI3K/Akt pathway are essential components of intracellular signal transmission. These pathways are involved in a variety of biological processes, including apoptosis and autophagy, among others. Previous research has demonstrated that doxorubicin’s ability to damage the heart can be mitigated by either activating the Akt pathway and downstream signaling molecules, such as Bad and the mammalian target of rapamycin (mTOR), or by inhibiting the MAPK p38 pathway. Therefore, one of the mechanisms by which Rg1 prevents doxorubicin-induced cardiac toxicity is the activation of the Erk and Akt pathways.

It is well established that the relative amounts of anti- and pro-apoptotic proteins regulate both cell survival and apoptosis. One of the primary proteins that prevent cells from going through the apoptosis process is called Bcl-2, and it is a member of the Bcl-2 family. The oligomerization of Bax can be stopped by combining it with a protein that promotes apoptosis. The oligomeric form of Bax is what causes the integrity of the mitochondrial membrane to be compromised and leads to the release of Cyt. C (104). In addition to this, doxorubicin reduces the ratio of Bcl-2 to Bax, which is an important component in the process of apoptosis (105). The oral administration of Rg1 elevate the ratio of Bcl-2 to Bax, which inhibited the mitochondrial release of Cyt. C and, as a result, reduced the amount of cardiac apoptosis that was induced by doxorubicin. This suggests that the imbalance between pro-apoptotic and anti-apoptotic proteins of the Bcl-2 family is another important mechanism by which Rg1 inhibits the cardiac toxicity caused by doxorubicin (26, 106).

### 4.4. Future perspective

Cancer is one of the most important causes of death in the world. So far, different cancer treatment methods have been suggested and are clinically used. Chemotherapy is one of these cases, which is widely used as a sole treatment or in combination with other treatments. Chemotherapy has many side effects that induce limitations in this treatment; one of the most important side effects is cardiac side effects (45, 94). As shown in Table 1, exposure to chemotherapy drugs can lead to various changes in the heart. In such situations, strategies should be provided to minimize the effect of chemotherapy-induced cardiac complications. This systematic review investigated the cardiac side effects of chemotherapy drugs at the levels of cellular and animal reduced by ginseng derivatives; however, a meta-analysis study is suggested in this line. These preclinical studies demonstrated that the administration of ginseng derivatives with chemotherapy drugs reduced oxidative stress, blocked the signaling pathway of inflammation and apoptosis, and led to the reduction of heart damage. Based on the results of this present preclinical study, it can be concluded that ginseng divagates are effective in cardiac disorders caused by exposure to chemotherapy drugs. Still, no sufficient human data is available so far. Recently, a clinical study in woman patients with non-metastatic breast cancer undergoing chemotherapy with doxorubicin reported that ginseng supplementation might protect against cardiac dysfunction associated with doxorubicin-induced early cancer therapeutics-related cardiac dysfunction and early decline in left ventricular ejection fraction in breast cancer patients (107). But it also needs further investigation on more patients and multiple cancers to prove or disprove this hypothesis.

## 5. Conclusion

The findings of this systematic study show that co-administration of ginseng with doxorubicin or cisplatin improves the biochemical and histopathological changes resulting from their use through anti-inflammatory, anti-oxidant, and anti-apoptotic mechanisms. Moreover, it reduces heart weight and the risk of heart toxicity from chemotherapy and ultimately reduces mortality. Therefore, according to this study of non-clinical studies, co-treatment with ginseng can reduces tolerability to chemotherapy agents which need to be proven and concluded more strongly with more studies, especially in the human field.

## Data availability statement

The original contributions presented in the study are included in the article/supplementary material, further inquiries can be directed to the corresponding authors.

## Author contributions

AMJ and AA: conceptualization, writing – original draft, and writing – review and editing. HH-A and RN: conceptualization, roles, writing – original draft, and writing – review and editing. All authors contributed to the article and approved the submitted version.

## Conflict of interest

The authors declare that the research was conducted in the absence of any commercial or financial relationships that could be construed as a potential conflict of interest.

## Publisher’s note

All claims expressed in this article are solely those of the authors and do not necessarily represent those of their affiliated organizations, or those of the publisher, the editors and the reviewers. Any product that may be evaluated in this article, or claim that may be made by its manufacturer, is not guaranteed or endorsed by the publisher.
