# Supplementary material for: The role of ginseng derivatives against chemotherapy-induced cardiotoxicity: A systematic review of non-clinical studies
Source: Front Cardiovasc Med. 2023 Feb 9;10:1022360. doi: 10.3389/fcvm.2023.1022360 (PMC9946988; doi:10.3389/fcvm.2023.1022360)
Supplement: Supplementary file 1 [file Table_1.docx]

| Table: The search keywords of the present study | | | | |
| --- | --- | --- | --- | --- |
| Shinseng keywords | AND | Chemotherapy keywords | AND | Heart (toxicity) keywords |
| Ginsenosides OR Sanchinosides OR Panaxosides OR Ginsenoside OR Panax OR "ginseng saponin" OR ginsenine OR ginsenol OR panaxagin OR protopanaxatriol OR protopanaxadiol OR protopanaxa OR panaxoside OR gingilone OR ginsenoside OR "ren shen" OR shinseng OR Ninjin OR Ninjins OR Renshen OR Renshens OR Shinseng OR Shinsengs OR "Jen Shen" OR "Jen Shens" OR "Shen, Jen" OR Ginseng OR Ginsengs OR Schinseng OR Schinsengs OR "Korean Red Ginseng" OR "Ginseng, Korean Red" OR "Korean Red Ginsengs" OR "Korean Ginsengs" OR "Korean Ginseng" OR "Red Ginseng, Korean" OR "Ginseng, Korean" OR "Panax ginseng" | AND | Chemotherapy OR Adriamycin OR Doxorubicin OR Eloxatin OR Bleomycin OR 4-demethoxydaunorubicin OR Idarubicin OR 4-demethoxydaunorubicin OR Cisplatin OR Carboplatin OR Cyclophosphamide OR Cytophosphane OR Paraplatin OR Daunorubicin OR Daunomycin OR Oxaliplatin OR Methotrexate OR Amethopterin OR Taxotere OR Etoposide OR Xeloda OR Mitomycin OR Vinorelbine OR Carmustine OR Melphalan OR Etopophos OR Procarbazine OR Epirubicin OR Vinblastine OR Navelbine OR Methotrexate OR Amethopterin OR Docetaxel OR Vincristine OR Mustine OR Fluorouracil OR Adrucil OR Embikhin OR Oxaliplatin OR Cytosine OR Capecitabine OR Paclitaxel OR Taxol OR Mechlorethamine OR Eloxatin OR Cytarabine OR Irinotecan | AND | Heart OR Myocytes OR Cardiotoxicity OR Cardiomyocyte OR Cardiomyopathies OR Myocardial OR Cardiomyopathy OR Myocardium OR Cardiopathy OR Cardiotoxicity OR Cardiopathic OR Cardi* |
